# Preclinical Parkinson’s Disease Models for Non-Motor Symptoms: Research Recommendations from a Systematic Review

**DOI:** 10.3390/life15071034

**Published:** 2025-06-28

**Authors:** Mariana Lara Zambetta, Elayne Borges Fernandes, Allison Kim, Thiago Luiz Russo, Anna Carolyna Gianlorenço

**Affiliations:** 1 Neuroscience and Neurological Rehabilitation Laboratory, Physical Therapy Department, Federal University of Sao Carlos, Sao Carlos 13565-905, Brazil; mariana.zambeta@hotmail.com (M.L.Z.); elayne@estudante.ufscar.br (E.B.F.); 2 Department of Physical Therapy, Federal University of Sao Carlos, Sao Carlos 13565-905, Brazil; russo@ufscar.br; 3 The Research Institute, Irvine, CA 92618, USA; 4 Spaulding Neuromodulation Center, Harvard Medical School, Cambridge, MA 02138, USA

**Keywords:** Parkinson’s disease, animal model, non-motor symptoms

## Abstract

Parkinson’s disease (PD) is a progressive neurodegenerative disorder primarily characterized by motor impairments resulting from dopaminergic neuron degeneration in the substantia nigra. However, PD is increasingly recognized as a multisystem disorder, where non-motor symptoms such as cognitive impairment, mood disturbances, sleep disorders, and autonomic dysfunction significantly impact patients’ quality of life. These non-motor symptoms often exhibit poor responsiveness to traditional dopaminergic therapies, underscoring a critical gap in current treatment strategies. Our systematic review investigates established methods of PD induction in rodent models and evaluates the methodologies used to assess non-motor symptoms. The review was performed following the Preferred Reporting Items for Systematic Reviews and Meta-Analysis (PRISMA) and the Cochrane handbook. Thirty-two studies from 832 articles were included. The studies were characterized by MPTP, 6-OHDA, and rotenone. Our results indicate that there was considerable heterogeneity in behavioral and motor tests, which poses challenges for data comparability and highlights the lack of consensus regarding the most appropriate modeling strategies for specific PD-related behavioral outcomes. All three models demonstrated behavioral changes consistent with dopaminergic impairment when compared to control groups. MPTP-induced models showed significant non-motor deficits across various tests, except in social recognition and novelty-suppressed feeding. The 6-OHDA model consistently produced non-motor impairments, supporting its utility in replicating PD-like neurotoxicity. Rotenone-treated animals exhibited reduced social interaction, decreased sucrose preference, and increased immobility in behavioral assays, further supporting its validity. Overall, our findings indicate that these neurotoxin-based models are effective in reproducing non-motor symptoms of PD, though methodological heterogeneity highlights the need for greater standardization in future preclinical research.

## 1. Introduction

Parkinson’s disease (PD) is a neurodegenerative and progressive disorder, first described by James Parkinson in 1817. It is estimated that 0.3% of the general population and 1% of the global population over the age of 65 have the disease, with its incidence increasing 5 to 10 times between the sixth and ninth decades of life [1]. From a pathophysiological perspective, PD may be triggered by a range of factors, with mitochondrial dysfunction, oxidative stress, protein aggregation such as α-synuclein, exacerbation of microglial inflammation, impaired autophagy, pathogenic genetic mutations, and neuroinflammation among the main contributors [2]. The fact is that the common final pathway of pathogenic mechanisms damaging the substantia nigra neurons leads to neuronal death.

Although classically defined as a motor disorder associated with dopaminergic neurodegeneration of the striatum, Parkinson’s disease (PD) also involves non-motor symptoms [3] such as neuropsychological, neuropsychiatric, autonomic, sleep, and pain disorders, which may precede the onset of motor symptoms [4]. Approximately 80% of PD patients, after 15 to 20 years of disease progression, experience cognitive impairment and dementia, and 50% develop depressive disorders. This is because PD extends beyond dopaminergic impairment, also affecting serotonergic and noradrenergic neurons, suggesting that dysregulation of neural circuits associated with mood regulation and reward systems is likely a cause of depressive disorders [5], resulting in a significant social, economic, and emotional burden on individuals diagnosed with the disease [1].

Since the 1960s, the use of L-DOPA has remained the primary pharmacological treatment for Parkinson’s disease to control both motor and non-motor symptoms. However, in the long term, L-DOPA administration leads to issues such as motor and non-motor fluctuations, along with a range of dyskinesias [6]. To date, no treatment is available to halt or slow the progression of PD [7], highlighting the need for studies that further explore its mechanisms and develop better therapeutic strategies.

Although no animal model currently in use can fully reproduce the behavioral and pathological characteristics observed in the typical form of PD in humans [7], animal studies have proven essential in understanding the disease’s pathological mechanisms. These models serve as valuable tools for investigating dopaminergic cell vulnerability, potentially mirroring clinical observations and providing reliable and reproducible phenotypes [8,9].

However, when it comes to studies that aim to investigate motor and non-motor behaviors in PD, the literature presents controversies and a lack of consensus regarding which best design should be used for each purpose. According to Vieira et al. (2019) [10], some authors [11] argue that anxiety is not a common feature observed in the 6-OHDA model, while other researchers [12] reported anxiogenic effects using the same model, although with different protocols.

Considering the fact that non-motor symptoms do not respond significantly to dopaminergic medication and are a challenge for the clinical treatment of PD, and the need to standardize research protocols for disease models, this systematic review aims to outline the main methods used to induce Parkinson’s disease in rodent models found in the literature, as well as their evaluation methodology for non-motor symptoms of the disease, in order to guide future studies and the best protocol for each purpose.

## 2. Methods

This review was registered at PROSPERO under the registration number CRD42024589595 and followed the guidelines of the Preferred Reporting Items for Systematic Reviews and Meta-Analysis (PRISMA) “https://www.prisma-statement.org/ (accessed on 15 January 2025)” and the Cochrane handbook for systematic reviews “https://training.cochrane.org/handbook (accessed on 15 January 2025)”. The literature search was performed in PubMed/Medline, Scopus and Web of Science databases until March 2025.

### 2.1. Eligibility Criteria

We used the following inclusion criteria: (1) preclinical animal studies; (2) using rodents; (3) using neurotoxins to mimic the disease; (4) that had a control group; (5) assessing non-motor symptoms; (6) from the last 12 years; (7) published in English. We excluded (1) other models; (2) other languages; (3) reviews and human studies.

### 2.2. Information Sources and Selection Procedure

Two independent researchers conducted electronic searches in PubMed, Scopus, and Web of Science from January to March 2025. All database results were merged using the web-based tool Rayyan “https://www.rayyan.ai/ (accessed on 24 March 2025)” to first screen articles based on titles and abstracts and to automatically exclude duplicates at this stage. After that, the researchers reviewed the application of the criteria and extracted the data. They checked the data for consistency and resolved disagreements by mutual consensus.

### 2.3. Outcomes

The results of interest were the non-motor symptoms of PD evaluated by the researchers through validated behavioral tests, such as the sucrose preference test, tail suspension test, forced swimming test, novel object recognition task, T-maze or Y-maze test, elevated plus maze test, step-through passive avoidance, Morris water maze test, among others.

### 2.4. Search Strategy

Broad key terms were employed, not restricting them to titles and abstracts. The search strings included the terms “Parkinson’s disease”, “animal model”, “behavior”, “motor activity OR non-motor activity”, and “therapeutics” adapted to each database (Table 1). Whenever available, filters were applied to avoid unnecessary duplicate data retrieval (e.g., clinical trial, systematic review, date, etc.).

### 2.5. Data Synthesis

All included articles were descriptively summarized in a standardized table, including the following information: author/year, sample (sample size and lineage), intervention elements (drug and dosage (dosage indicates the number of doses on consecutive days)), recovery time (period until the first behavioral test), measurement (behavioral tests) and significant results.

### 2.6. Methodological Quality and Confidence in the Evidence

The SYRCLE tool was adopted to assess the risk of bias/methodological quality of the included studies. The resulting RoB tool for animal studies contains 10 domains: (1) sequence generation; (2) baseline characteristics; (3) allocation concealment; (4) random housing; (5) blinding; (6) random outcome assessment; (7) blinding; (8) incomplete outcome data; (9) selective outcome reporting; and (10) other sources of bias [13]. Two independent investigators assessed the papers. This tool classifies the level of confidence in the evidence as low risk of bias (+), high risk of bias (−), and unclear risk of bias (?) based on study characteristics, such as selection bias, performance bias, detection bias, attrition bias, reporting bias and other biases.

## 3. Results

**Figure 1:**
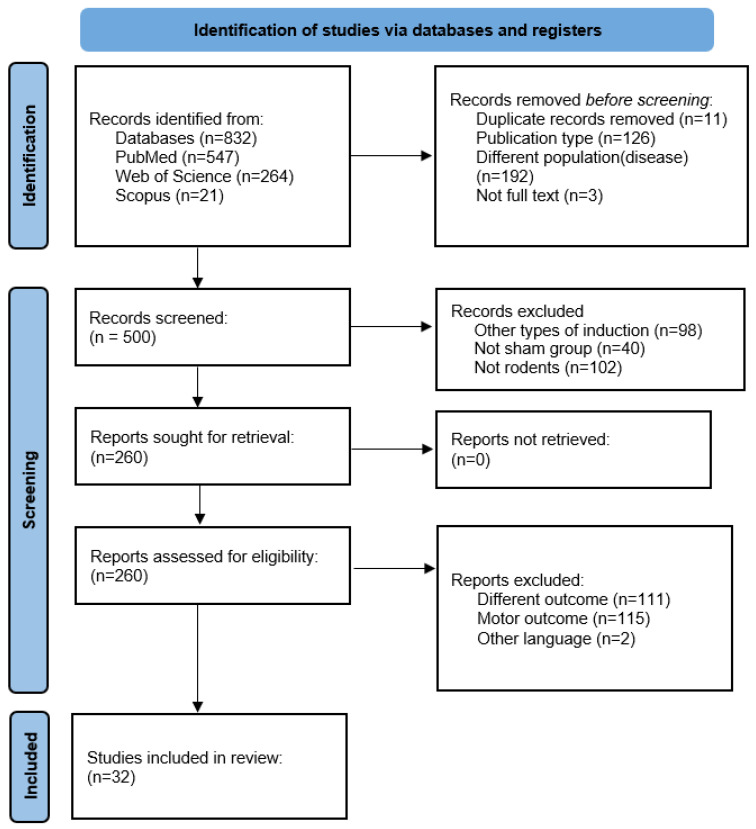
PRISMA flow diagram illustrating the systematic search across the databases: experimental studies.

A total of 832 articles were found in the initial search. After the selection process and application of the eligibility criteria, we ended up with 32 studies in the systematic review. The selection process, including the reasons for exclusion of records, is described in Figure 1.

### 3.1. Overall Characteristics of the Studies

**Figure 2:**
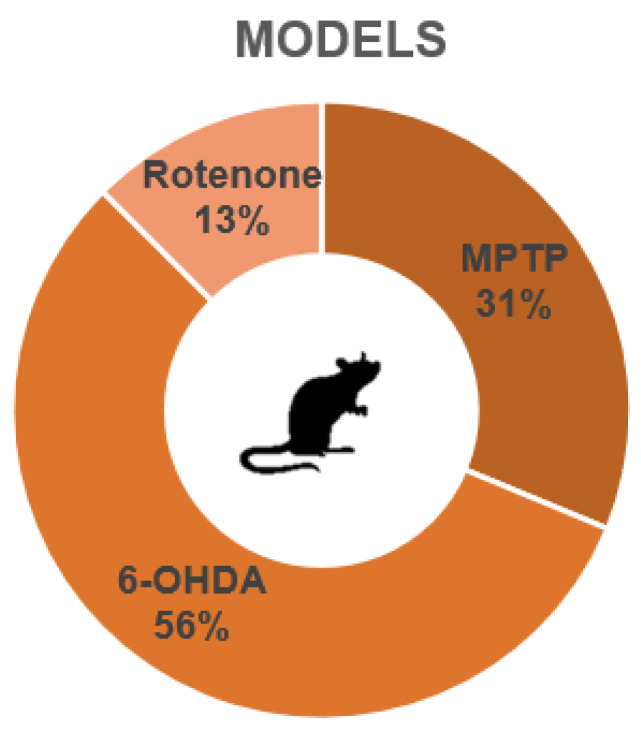
Representation of PD models.

**Figure 3:**
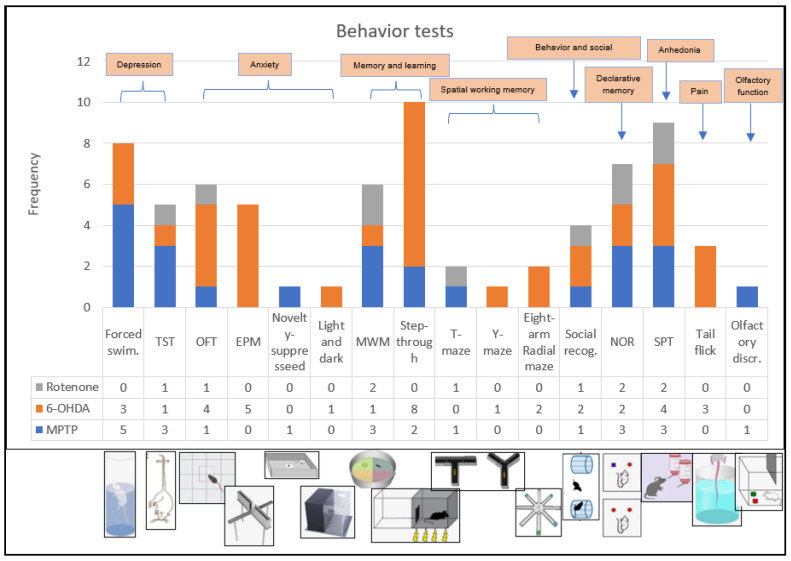
General overview of the distribution of behavioral tests in relation to each model of Parkinson’s disease induction: forced swimming test and TST were used to assess depression; OFT, EPM, novelty-suppressed (novelty-suppressed feeding) and light and dark test were used to assess anxiety; MWM and step-through (step-through passive avoidance test) were used to assess memory and learning; T-maze, Y-maze and eight-arm radial maze were used to assess spatial and working memory; social recognition was used to assess behavior and social memory; NOR was used to assess declarative memory; SPT was used to assess anhedonia; tail flick was used to assess pain perception; and olfactory discrimination was used to assess olfactory function.

A descriptive characterization of the studies is summarized in Table 2, Table 3 and Table 4. We included 32 experimental studies in this systematic review. The preclinical models of Parkinson’s disease included in this review applied three main compounds to induce PD symptoms: 1-methyl-4-phenyl-1,2,3,6-tetrahydropyridine (MPTP), 6-hydroxydopamine (6-OHDA), and rotenone. Ten of these studies (representing 31% of these studies) used MPTP (Table 2); eighteen studies (56%) used 6-OHDA (Table 3); and four studies (13%) used rotenone (Table 4) (Figure 2). We were able to observe a great heterogeneity in the sampling of the studies, both with regard to the lineage of rodents, the dosage of substances that mimic PD, and the recovery time of the animals, as well as in the behavioral tests chosen by each of the studies. Figure 3 shows an overview of the models and behavioral tests found in this study.

### 3.2. Methodologies: MPTP

MPTP is a neurotoxin commonly used to model Parkinson’s disease in animals, particularly in primates and rodents. It induces a parkinsonian syndrome that closely mimics the clinical and pathological features of Parkinson’s disease. Once in the brain, MPTP is metabolized into MPP+, a toxic compound that selectively targets and damages dopaminergic neurons in the substantia nigra. This neuronal loss leads to the hallmark motor symptoms of parkinsonism, making MPTP a valuable tool for studying disease mechanisms and testing potential therapies. The characteristics of the included MPTP studies can be seen in Table 2. In our review, seven of the MPTP studies were performed in C57BL/6 mice, while the other three studies used Wistar rats; all male. The sample size was between a minimum of twenty-four and a maximum of sixty-three animals, ranging from 5 to 13 animals per group. We can also observe a variation in the dosage and route of administration of MPTP between the studies. Seven of these studies performed intraperitoneal application: ranging from 35 mg/kg applied for twenty-eight consecutive days [14]; 30 mg/kg being applied for seven days [15,19,21]; 7 mg/mL applied for five days [16]; and 25 mg/kg applied for five consecutive days [18,22]. The three remaining studies varied between intranasal, intravenous and infusion application: the first study performed intranasal application with 0.1 mg/nostril for one, two and three days [17]; the second study performed intranasal + intravenous application with 1mg/nostril + 20mg/mL intravenous in just one day [23]; and the third study performed infusion application in the substantia nigra with 1 μmol in 2 μL of saline of MPTP also in just one day [20].

After the PD induction period, the animals were subjected to a recovery period until they could perform the behavioral tests. This period is necessary both to allow recovery and for the toxin-induced lesion to stabilize. We were also able to observe variability in this time between the last day of substance administration and the first day of behavioral tests. Three studies performed the tests seven days after the end of modeling [15,19,21]; one study performed them from 5 to 9 days [16]; another study performed the behavioral tests at three different times; seven, fourteen, and twenty-one days [17]; two other studies began the tests twenty-eight days after the end of modeling [18,22]; two other studies performed each of their different tests on different days, ranging from eight to fourteen days for one study [20] and seven, fourteen, and twenty-one for the other study [23]; and one study performed the tests fifty-six days after the end of modeling [14].

**Figure 4:**
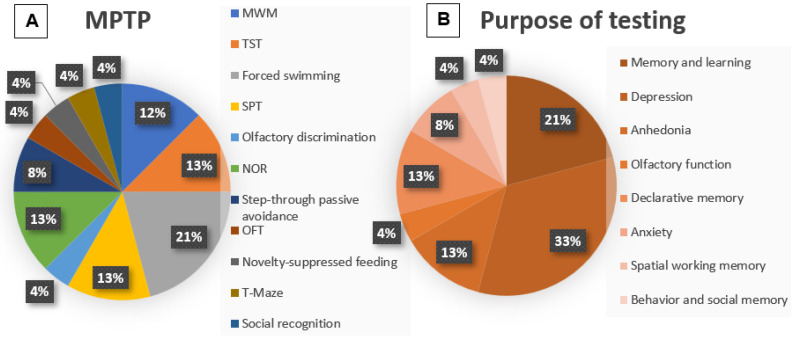
(**A**) Graphical representation of the frequency of the tests used in MPTP studies. This figure shows the percentage representation of the reproduction percentage of each test among the included MPTP studies: MWM (12%); TST (13%); forced swimming test (21%); SPT (13%); olfactory discrimination (4%); NOR (13%); step-through passive avoidance test (8%); OFT (4%); novelty-suppressed feeding test (4%); T-maze test (4%); and social recognition test (4%). (**B**) Graphical representation of the purpose of the tests used in MPTP studies. This figure shows, in percentage, the behavioral objectives evaluated in the MPTP studies: memory and learning (21%); depression (33%); anhedonia (13%); olfactory discrimination (4%); declarative memory (13%); anxiety (8%); spatial and working memory (4%); and social behavior and memory (4%).

Regarding the behavioral tests used, we also observed heterogeneity in the choice in each study. It is important to emphasize that some of the studies included here also investigated motor behavior through the performance of motor tests. However, according to the objectives of this review, we will analyze only non-motor skills. Each study performed more than one behavioral test (Figure 4A); thus, the Morris water maze test (MWM) was reproduced in three studies (12%) [14,21,23]; the tail suspension test (TST) was also reproduced in three studies (13%) [15,16,19]; the forced swimming test in five studies (21%) [15,16,17,19,23]; the sucrose preference test (SPT) in three studies (13%) [16,19,23]; the olfactory discrimination test in one study (4%) [17]; the novel object recognition task (NOR) in three studies (13%) [18,20,22]; the step-through passive avoidance test in two studies (8%) [18,22]; the open field test (OFT) with non-motor parameters such as time spent in the center of the test box was reproduced in one study (4%) [19]; novelty-suppressed feeding also in one study (4%) [19]; the T-maze or Y-maze test in one study (4%) [20]; and, finally, the social recognition test was reproduced in only one study (4%) [23].

### 3.3. Outcome: MPTP

In the study by [23] no significant differences were found in the SPT and MWM tests in the MPTP groups when compared to the control groups. Furthermore, in the study by [19] no significant differences were observed in the OFT and novelty-suppressed feeding tests. The remaining tests performed in both studies were favorable to modeling.

In the other studies, we observed significant differences between the MPTP and control groups, which is indicative of dopaminergic impairment caused by the toxin. The following results were found in the MPTP groups when compared to the control: increased escape latency in the MWM [14,21]; increased immobility time during the TST [15,19]; increased immobility time during the forced swimming test [15,16,17,19,23]; decreased sucrose preference in the SPT [16,19]; olfactory impairment in the olfactory discrimination test [17]; decreased or absent object discrimination in the NOR [18,20,22]; decreased latency to enter the dark compartment in the step-through passive avoidance [18,22]; and memory deficit in the T-maze test [20].

### 3.4. Methodologies: 6-OHDA

Briefly, to induce PD by neurotoxin infusion, animals are anesthetized, positioned in a stereotaxic frame, and subjected to striatal injections of 6-OHDA. Unilateral or bilateral lesions are performed by injecting 6-OHDA hydrochloride using a Hamilton syringe coupled to an infusion pump. The neurotoxin is administered according to the stereotaxic coordinates defined for each study. After each injection, the needle is left in place for a few minutes for diffusion and to prevent reflux.

As in the MPTP studies, we can also observe heterogeneity in the methodology used in each 6-OHDA study. The characteristics of the included 6-OHDA studies can be seen in Table 3.The great majority of the 6-OHDA studies (n = 15) were performed in Wistar rats, one in Sprague–Dawley rats, and two in C57Bl/6 mice; all males. The sample size was from a minimum of twenty-four and to a maximum of seventy-two animals, ranging from six to sixteen animals per group. Regarding the neurotoxin dosages: four studies performed 6-OHDA infusion in the right medial forebrain bundle (MFB) with doses ranging from 20 µg/4 µL [24]; 24 μg [25]; 16 μg/2 μL [27]; 2 μL [33]. Five other studies performed the infusion of 6-OHDA into the left MFB, in turn, with doses ranging from 2 µL/min [26]; 16 μg/4 μL [28,29]; 3 μg/μ [34]; and 6.5 μg [36]. One study describes only having performed infusion of the neurotoxin into the MFB [37]. Another study performed the infusion of 1 μL of 6-OHDA into the substantia nigra pars compacta [30]. Two other studies describe only the infusion of 6 μg/μ [31] and 10 μg [41] into the substantia nigra. Other studies performed the infusion of 200 μg/5 μL intracerebroventricular/third ventricle [32]; 12 μg into the dorsal striatum [35,39]; 18 μg/3 μL intrastriatal [38]; and 8 μg 6-OHDA unilaterally in the terminal region of the striatum [40]. Furthermore, only five of the eighteen studies performed the application of desipramine minutes before the infusion of the neurotoxin, varying between 25 mg/kg [24,32,41] and 15 mg/kg [27] in the dosage.

The recovery time of the animals after the infusion, until they were submitted to the behavioral tests, also showed great variability. Here, thirteen of the eighteen studies performed an inclusion test of the animals, with the apomorphine-induced rotational behavior test performed in eleven studies and D-amphetamine-induced rotation performed in two studies. This time varied between one week, two weeks, three weeks, four weeks and six weeks after the injury for the apomorphine-induced rotational behavior test and one, three and four weeks for the D-amphetamine-induced rotation. We do not analyze rotational behavior here.

**Figure 5:**
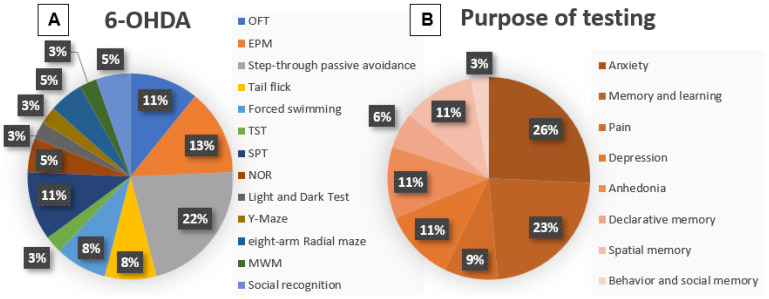
(**A**) Graphical representation of the frequency of the tests used in 6-OHDA studies. This figure shows the percentage representation of the reproduction percentage of each test among the included 6-OHDA studies: OFT (11%); EPM (13%); step-through passive avoidance test (22%); tail flick test (8%); forced swimming test (8%); TST (3%); SPT (11%); NOR (5%); light and dark test (3%); Y-maze test (3%); eight-arm radial maze test (5%); MWM (3%); and social recognition (5%). (**B**) Graphical representation of the purpose of the tests used in 6-OHDA studies. This figure shows the percentage representation of the reproduction percentage of each test among the included 6-OHDA studies: anxiety (26%); memory and learning (23%); pain perception (9%); depression (11%); anhedonia (11%); declarative memory (6%); spatial and working memory (11%); and behavior and social memory (3%).

Non-motor behavioral tests were performed: one [30,35,40], two [26,30,32,35,38,39,41], three [24,31,33,36,37], four [25], six [28,29] and seven [34] weeks; and four months [27] after the neurotoxic injury, varying between studies. Each of the studies performed more than one test (Figure 5A); thus, the OFT was reproduced in four studies (11%) [24,25,26,27], the elevated plus maze test (EPM) was reproduced in five studies (13%) [24,25,27,35,39], the step-through passive avoidance in eight studies (22%) [24,26,28,29,32,34,38,41], the tail flick test in three studies (8%) [24,27,29], the forced swimming test in three studies (8%) [25,33,40], the TST in only one study (3%) [25], the SPT in four of the studies (11%) [30,34,35,39], NOR in two studies (5%) [31,39], light and dark in one study (3%) [33], Y-maze (3%) [41], and eight arm radial test maze in three studies (5%) [36,37], MWM in one study (3%) [38], and finally, social interaction test in two studies (5%) [33,39].

### 3.5. Outcome 6-OHDA

The study by [25] found no significant differences between the 6-OHDA and control groups in the OFT, EPM, forced swimming test, and TST. In addition, the study by [36], also found no significant difference in the eight-arm radial maze. The other studies presented results favorable to the modeling of PD, which may indicate dopaminergic impairment due to neurotoxicity caused by 6-OHDA.

The following results were observed in the 6-OHDA groups when compared to the control groups: increased immobility time [24], increased time spent in the central zone [26], and reduced grooming frequency [27] in the lesion group in the OFT; decreased number of entries and time spent in the open arms in the EPM [24,27,35,39]; decreased latency to pass through or enter the dark compartment in the injury groups in the step-through passive avoidance test [24,26,28,29,32,34,38,41]; decreased nociceptive response time in the tail flick test [24,27,29]; increased time spent immobile in the water during the forced swimming test [33,40]; decreased sucrose preference [30,35,39] and decreased total fluid consumption [34] in the SPT; decreased discrimination index between two objects in the NOR [31,39]; reduced time spent in the light chamber in the light and dark test [33]; impaired short-term memory [37] and decreased percentage of spontaneous alternations [41] in the eight arm radial test maze and Y-maze, respectively; increased escape latency time in the MWM [38]; and reduced time spent with rats of the same species [33] and impaired results [39] in the social interaction test.

### 3.6. Methodologies: Rotenone

The rotenone model of Parkinson’s disease (PD) is a well-established and widely utilized experimental model, particularly in rodents, that mimics many of the key pathological and behavioral features of PD. Rotenone is a naturally occurring pesticide that functions as a potent inhibitor of mitochondrial complex I, a critical component of the electron transport chain. Chronic systemic administration of rotenone leads to mitochondrial dysfunction, oxidative stress, and selective degeneration of dopaminergic neurons in the substantia nigra pars compacta—hallmarks of Parkinson’s disease pathology.

This model replicates not only the loss of nigrostriatal dopaminergic neurons but also the formation of intracellular protein aggregates resembling Lewy bodies, a pathological signature of PD. Additionally, animals exposed to rotenone exhibit motor deficits, such as bradykinesia and postural instability, that parallel the symptoms seen in human patients.

The rotenone model is particularly valuable for investigating the role of mitochondrial dysfunction, oxidative damage, neuroinflammation, and environmental toxins in PD pathogenesis. It also provides a platform for evaluating neuroprotective strategies and testing potential therapeutic interventions aimed at halting or reversing disease progression.

The characteristics of the included Rotenone studies can be seen in Table 4. Of the four rotenone studies, three used Wistar rats and one study used C57BL/6; all male. The sample size ranged from thirty, forty and one hundred and twenty-six animals, with 6 to 12 per group. Rotenone was administered to the animals as follows: 1.5 mg/kg rotenone subcutaneously for eight days [42,44]; 2.5 mg/kg intraperitoneally for fourteen days [43]; and 3 mg/kg subcutaneously for five days [45].

**Figure 6:**
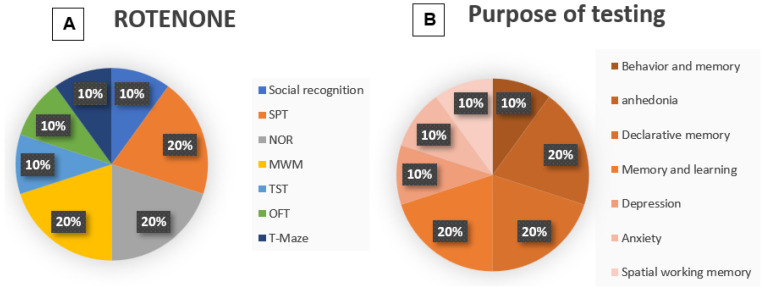
(**A**) Graphical representation of the frequency of the tests used in rotenone studies. This figure shows the percentage representation of the reproduction percentage of each test among the included Rotenone studies: Social recognition (10%); SPT (20%); NOR (20%); MWM (20%); TST (10%); OFT (10%); and T-maze test (10%). (**B**) Graphical representation of the purpose of the tests used in rotenone studies. This figure shows the percentage representation of the reproduction percentage of each test among the included rotenone studies: behavior and memory (10%); anhedonia (20%); declarative memory (20%); memory and learning (10%); depression (10%); anxiety (10%); and spatial and working memory (10%).

The animals were subjected to behavioral tests one day [45], two weeks [42], three weeks [44], and four weeks [43] after the application of rotenone. Each of the studies performed more than one test (Figure 6A); thus, the social interaction [42], T-maze test (10%) [45], TST (10%) [43], and OFT (10%) [45] were reproduced only once, while the SPT (20%) [42,43], NOR (20%) [42,44], and MWM (20%) [42,44] were reproduced twice each.

### 3.7. Outcome Rotenone

Here, the results of the rotenone groups also indicate dopaminergic impairment caused by rotenone. The following results were observed in the rotenone groups compared to the control groups: reduction in the number of interactions in the social interaction test [42]; reduction in sucrose preference in the SPT [42,43]; decrease in novel object preference in the NOR [42,44]; increase in escape latency in the MWM [42,44]; increase in immobility time during the TST [43]; increase in immobility time during the OFT [45]; and decrease in the percentage of memory retention in the T-maze test [45].

### 3.8. Quality Assessment

**Figure 7:**
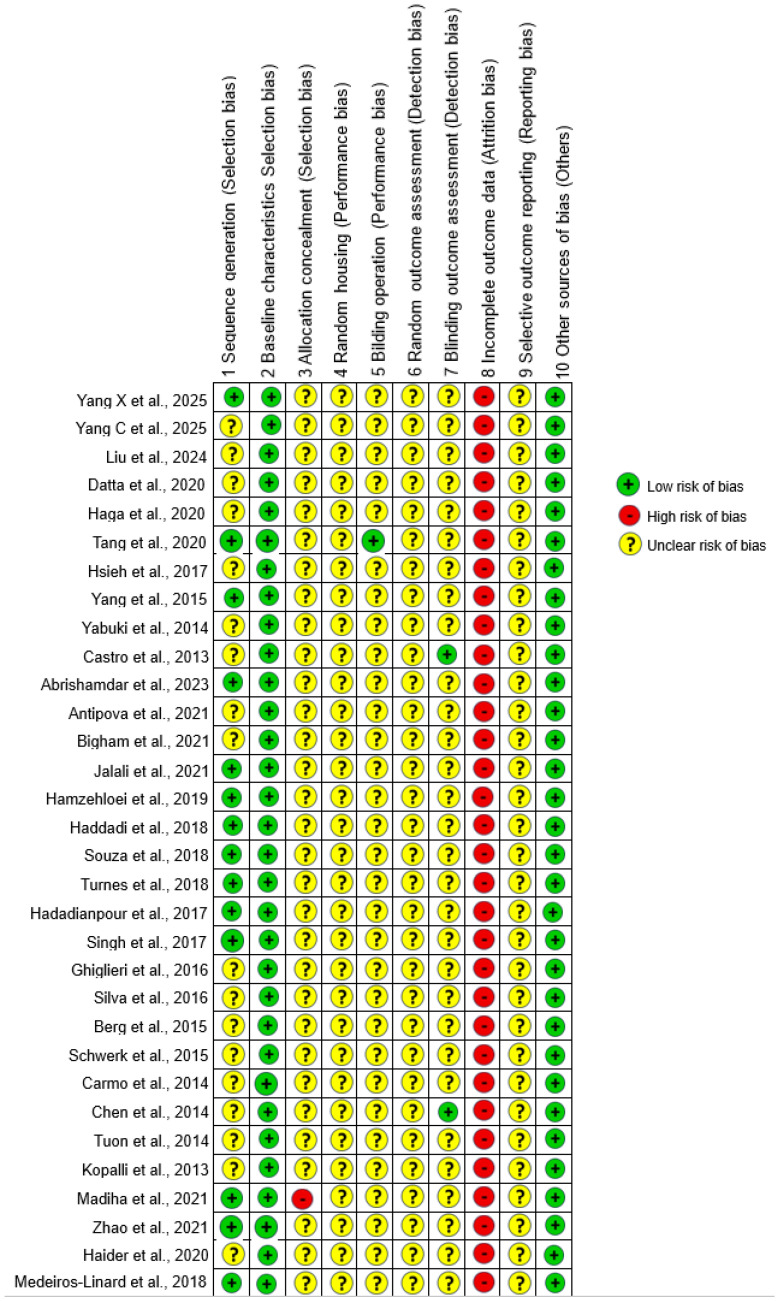
Overview of bias assessment in included studies using the SYRCLE risk of bias tool for animal studies [14,15,16,17,18,19,20,21,22,23,24,25,26,27,28,29,30,31,32,33,34,35,36,37,38,39,40,41,42,43,44,45].

Figure 7 shows the complete assessment result, using the Syrcle Assessment Tool. All studies presented low risk of bias only in domains 2 and 10, and high risk of bias only in domain 8. Of the thirty-two articles evaluated, only fourteen reported randomization of groups (domain 1) [14,19,21,24,27,28,29,30,31,32,33,42,43,45], only one study reported blinding of caretakers and/or researchers regarding the intervention that each animal received during the experiment (domain 5) [19], and only two studies reported blinding of evaluators (domain 7) [23,39], thus presenting low risk of bias in their respective domains. In addition, one study makes it clear that it did not conceal the allocation sequence, showing high risk of bias in domain 3 [42]. The remaining studies that did not present such information in domains 1, 3, 5 and 7 scored unclear risk of bias. All studies showed unclear risk of bias in domains 4, 6 and 9, since the authors did not report information regarding the random housing of animals during the experiment, random selection of animals for evaluation of results, and selective reporting of results.

## 4. Discussion

In this systematic review, we aimed to evaluate established methods of inducing Parkinson’s disease in rodent models, as well as the methodologies used in the assessment of non-motor symptoms. Genetic models of PD, such as Parkin, PINK1 and DJ1, knock-in and knock-out models, viral overexpression models, α-synuclein protein propagation models and liposaccharide-induced models are also commonly found; however, in the present review, we included only environmental models: 1-methyl-4-phenyl-1,2,3,6-tetrahydropyridine (MPTP), 6-hydroxydopamine (6-OHDA), and rotenone, which lead to acute cell death with limited aggressive pathology, representing an advanced stage of the disease [46]. All three compounds showed differences from control groups, indicating dopaminergic impairment, indicating that such established methods of inducing Parkinson’s disease are effective.

### 4.1. Model-Specific Observations

#### 4.1.1. MPTP

MPTP is a byproduct of the synthetic heroin 1-methyl-4-phenyl-4-propionoxypiperidine. It is a lipophilic compound and can cross the blood–brain barrier into the brain and be rapidly converted to the toxic metabolite 1-methyl-4-phenylpyridinium or MPP+ by monoamine oxidase-B. Thus, MPP+ is selectively taken up by dopaminergic neurons via dopamine transporters and inhibits complex I of the mitochondrial electron transport chain, causing parkinsonism [7].

In this review, the MPTP model was predominantly performed in C57BL/6 mice. Based on their genetic makeup, different strains of rodents may present varying degrees of dopaminergic loss when exposed to neurotoxins. Thus, the genetic characteristics of different strains may shape both behavioral traits, such as motor activity, anxiety and learning, as well as variable responses to injuries, neuroinflammatory responses and the way in which neurotoxins are metabolized or absorbed in each model, thus highlighting the importance of choosing the strain in each study according to the desired hypothesis.

In view of this, C57BL/6 mice have been shown to be the most sensitive strain to MPTP. Studies have reported that MPTP treatment in mice results in acute non-progressive loss of dopaminergic cells, accompanied by reductions in striatal dopamine, whereas rats and other strains are more resistant to its effects [46], corroborating our findings since seven of the ten studies included here used C57BL mice.

Furthermore, MPTP modeling demonstrated considerable variation in dosage and route of administration, ranging from chronic intraperitoneal injections to acute intranasal or intracerebral infusions. Of the ten studies on MPTP, seven used subchronic application and one used chronic application, corroborating other studies reporting that chronic and subacute MPTP models may be more appropriate for treatments, as they offer a larger window of early symptomatic stages when applying a drug [46].

Of the ten studies that used MPTP, only two of them presented controversial results in some of their behavioral tests. The study by [19], presented unfavorable results in two of the five tests performed, with no significant difference in OFT and novelty-suppressed feeding. The time given for lesion stability and recovery of the animals or other methodological issues may be possible factors for failure to identify non-motor symptoms. Despite this, the study concludes that it has obtained effectiveness in its MPTP modeling to evaluate depressive-type symptoms in animals, based on other tests and molecular analyses. The study by [23], presented unfavorable results in two of its four tests performed, with no significant differences in SPT and MWM. The authors justify that the low dose of MPTP and/or the route of administration used in the study (intranasal) may explain, in part, the discrepancy with previous findings. This reinforces the importance of standardizing research protocols in disease modeling, as well as their methodological standards.

#### 4.1.2. 6-OHDA

In summary, 6-OHDA is a lethal toxin that cannot cross the blood–brain barrier, so that central nervous system toxicity is only achieved by direct administration to the brain. The toxicity of 6-OHDA is related to its ability to produce free radicals and oxidative stress, similar to the effect of hydrogen peroxide, resulting in the loss of respiratory activity due to oxidative stress caused by free radicals. Initially, microglial stimulation and reactive oxygen species act synthetically with 6-OHDA and establish an adequate and early component of convinced cell death [7].

Here, the 6-OHDA model also exhibited diverse methodological strategies, particularly in terms of lesion site (e.g., medial forebrain bundle, striatum, substantia nigra) and dosages. Notably, the use of desipramine pretreatment to prevent noradrenergic toxicity was inconsistently applied, potentially influencing lesion specificity and behavioral outcomes. Despite these differences, most studies reported significant non-motor impairments, including cognitive deficits, anxious behavior, and anhedonia. Only two of eighteen studies found no significant differences in behavioral measures.

The study by [25] found a decrease in locomotor activity in the 6-OHDA group when compared to the control group in the OFT, but did not observe significant differences in parameters related to curiosity and exploration activity, such as time in the center, while the study by [26] found decreased time in the central zone in the 6-OHDA group when compared to the control. In the present review, of the four studies that used OFT for anxiety and depression purposes, one observed the time of immobility [20], two observed the time in the central zone [24,33], and one investigated the frequency of grooming [25], indicating low consistency for decision-making, since only four of the eighteen studies used OFT and despite this, they evaluated different parameters.

Furthermore, the study by [25], did not find significant differences between the lesion group and the control group in the EPM. The authors justify that DA depletion is necessary, but not sufficient by itself to induce an anxious phenotype, which contrasts with the studies by [24,27,35,39], which found a decrease in the number of entries and time spent in the open arms, thus favoring the lesion model. However, it is important to emphasize that the extent of the lesion and the specific targeting of the site are crucial for the validity of the model, and that each study, despite being similar, shows methodological differences between them, also making comparison difficult.

In the study by [36], no significant differences were found between the groups in the eight-arm radial maze test. The authors justify that the reduction in hippocampal neurogenesis caused by monohemispheric dopaminergic deprivation may not be sufficient to decrease learning or memory in this context. In addition to the study by Berg et al., 2015 [36], only one other study performed the eight-arm radial maze, also showing a low level of reproducibility and comparison in this model.

Regarding the choice of lineage in this model, studies show that depressive symptoms in animals are also commonly explored by liposaccharide modeling [46], which is used to induce inflammatory dopaminergic neurodegeneration. In another systematic review that studied the lipopolysaccharide-induced depression model in mice, it was observed that mice, as experimental subjects, have adequate sensitivity, validity and reliability to model depression, appearing to be more suitable than rats for application in models of depression induced by neuroinflammation [47].

In contrast, our review found that the 6-OHDA model was predominantly applied in Wistar rats, and despite some counterpoints, most of the studies included here were able to observe significant differences in behavioral measures in this model. The choice of rats for the 6-OHDA model is possibly due to the larger animal size for reproducibility of brain surgeries and microinjections, since richer behavioral patterns and higher translational correlation are commonly used in rats rather than mice [7].

#### 4.1.3. Rotenone

Rotenone, in turn, is a natural organic insecticide that can damage the electron transport chain of mitochondrial complex I, inducing mitochondrial dysfunction and leading to dopaminergic neuronal loss in the nigrostriatal pathway and the accumulation of reactive oxygen species [7].

The rotenone model, although less frequently used in the included studies, demonstrated a consistent pattern of non-motor symptom expression across all tests, particularly in the cognitive and affective domains. Given rotenone’s ability to mimic both dopaminergic degeneration and Lewy body-like pathology, its relevance to sporadic forms of PD is high. However, the small number of studies limits broad generalizability, and differences in route of administration (e.g., subcutaneous vs. intraperitoneal) and duration likely contributed to the variability in results.

#### 4.1.4. Comparison of Models and Concerns About Reproducibility

Characteristics of cognitive impairment and dementia in PD may be based on deficits in attention, executive function, visuospatial function, memory, and language [8]. In this review, we found assessments that primarily sought to assess symptoms such as depression, anxiety, spatial memory and learning, declarative memory, and spatial working memory, followed by other poorly reproduced symptoms such as pain perception, olfactory function, and anhedonia (Figure 3).

One of the most striking findings was the lack of standardization in behavioral testing. The wide variety of tests used to assess similar behavioral domains makes direct comparisons difficult. For example, depressive-like behavior has been assessed using the TST, the sucrose preference test, and the forced swim test—each with different sensitivities and conceptual underpinnings. Also in this context, the recovery time before the behavioral assessment varied markedly, probably affecting the degree of injury stabilization and neurochemical recovery, thus influencing the test results.

In this context, the recovery time before behavioral assessment varied markedly, which may affect the degree of lesion stabilization and neurochemical recovery. There are studies that support the idea that behavioral assessment of anxiety-like parameters in the 6-OHDA model should be performed 21 days after neurotoxin injection, in order to allow complete recovery of hypolocomotion [10]. Despite the variability observed here, most of the included studies began behavioral assessment two (in seven studies) and five (in five studies) weeks after neurotoxin infusion, which may indicate that this is a good estimate of the time to begin assessments.

In the present review, the most evaluated symptoms in the MPTP studies were depression (33%), through the forced swimming test and tail suspension test, and spatial memory and learning (21%) (Figure 4B). In the 6-OHDA model, the most evaluated symptoms were anxiety (26%), through OFT and EPM, and spatial memory and learning (23%) through the step-through passive avoidance test (Figure 5B). It is worth remembering that in the MPTP models, the majority used mice, while in the 6-OHDA models, the majority followed with Wistar rats.

These differences between the choice of tests in each model may be based not only on the base line, since genetic characteristics can influence behavior in a disease model, but also on the laboratory environment in which the studies were performed. Another systematic review, when investigating non-motor phenotypes in genetic animal models of PD, reports that when comparing two base line mice, it was identified that the main contributor to cognitive differences in these lineages were environmental effects and laboratory factors, and reinforces that optimizing the protocol for the specific base line and laboratory environment may represent a way to standardize different animal models and improve the reproducibility of the results [8].

In this review, the sex of the animals was not a criterion for inclusion or exclusion of the studies; however, 100% of the included articles used only male rodents. Preclinical studies of diseases, where sex is not a crucial factor for the research hypothesis, focus predominantly on male animals, and the main argument against the inclusion of females in these studies is based on the increase in hormonal variability during the estrous cycle, which could influence behavior, neuroanatomy, and physiology, among others [48].

However, a recent meta-analysis compared the variability of data from male and female rodents on behavioral measures of fear and anxiety and reported finding no evidence to support the claim that females are more variable than males, complementing previous reports that indicate no sex differences in other domains [10]. Although PD has a higher incidence in males, it is extremely important for translational research that preclinical studies be conducted in both sexes, raising an important discussion point for future studies.

### 4.2. Risk of Bias and Reporting Transparency

The quality assessment using the SYRCLE tool revealed concerning gaps in methodological transparency. Only a minority of studies reported randomization, blinding, or allocation concealment, which are fundamental for reducing experimental bias. Furthermore, the absence of information related to animal housing, selective reporting, and sample selection was common, indicating areas where reporting standards must be improved.

### 4.3. Conclusions and Future Directions

Based on the findings of this study, we can observe that 6-OHDA is the most widely used model in the literature to investigate non-motor symptoms of Parkinson’s disease, followed by the MPTP model. Furthermore, our results indicate that the choice of model, animal lineage, and behavioral tests should be considered before conducting any study, in addition to being based on the main research hypothesis, since such variables can directly interfere with the results. Here, we can conclude that the MPTP model investigated mainly depressive symptoms, while the 6-OHDA model investigated mainly anxiety symptoms, followed by spatial memory and learning in both models.

Our findings also underscore the critical need for methodological harmonization in preclinical PD research, particularly regarding the following: standardized protocols for toxin administration and recovery periods; consensus on behavioral test batteries that align with specific non-motor domains; rigorous reporting of experimental design and bias-reducing strategies.

Moreover, increasing the use of multimodal assessments that combine motor and non-motor evaluations could provide a more comprehensive understanding of PD symptomatology. Future research should also prioritize longitudinal designs and sex-balanced sampling, as all included studies used male animals, limiting translational relevance.

## Figures and Tables

**Table 1:** Comprehensive search strategy by database.

Database	String or Combined Search Terms
PubMed	((((parkinson’s disease) AND (animal model)) AND (behavior)) AND (motor activity OR no motor activity)) AND (therapeutics)
Scopus	(TITLE-ABS-KEY “parkinson’s disease”) AND TITLE-ABS-KEY (“animal model”) AND TITLE-ABS-KEY (behavior) AND TITLE-ABS-KEY (“motor activity”) OR TITLE-ABS-KEY (“not motor activity”) AND TITLE-ABS-KEY therapeutics))
Web of Science	(((((ALL = (parkinson’s disease)) AND ALL = (animal model)) AND ALL = (behavior)) AND ALL = (motor activity)) OR ALL = (no motor activity)) AND ALL = (therapeutics)

TITLE-ABS-KEY searches within the title, abstract, and keywords.

**Table 2:** Characterization of experimental studies included in the review: MPTP.

Author/ Year	Sample	Intervention Elements	Recovery Time	Measurement Non Motor Test	Significant Results
Yang X et al., 2025 [14]	n = ? male C57BL/6 mice	35 mg/kg MPTP i.p. 28 short	56 days	MWM	↑ time and movement distance
Yang C et al., 2025 [15]	n = 5/group male C57BL/6 mice	30 mg/kg MPTP i.p. 7 short	7 days	TST, FST and Coat score assay	↑ immobility TST and FST
Liu et al., 2024 [16]	n = 8/group male C57BL/6J mice	7 mg/mL MPTP i.p. 5 short	5 to 9 days	SPT, FST and TST	↑ immobility FST and TST. ↓ sucrose SPT
Datta et al., 2020 [17]	n = 8/group male Wistar, albino rats	0.1 mg/nostril MPTP. 1, 2 and 3 short	7–14–21 days	Olfactory discrimination and FST	↓ time spent in familiar compartments, ↑ immobility FST
Haga et al., 2020 [18]	n = 6/group male C57BL/6N mice	25 mg/kg MPTP i.p 5 short	28 days	NOR and step-through passive avoidance test	No ≠ NOR. ↓ step-through
Tang et al., 2020 [19]	n = 9–11/group male C57BL/6 mice	30 mg/kg MPTP i.p 7 short	1 day to include and 7 days other tests	OFT, TST, FST, SPT and novelty-suppressed feeding	↓ SPT. ↑ immobility TST and FST. No ≠ OFT and novelty- suppressed feeding
Hsieh et al., 2017 [20]	n = 13/group male Wistar rats	1 μmol in 2 μL of saline MPTP substantia nigra pars compacta. 1 short	8–9 T-maze test 14 = Object recognition test	T-maze test and NOR	↓ T-maze and NOR
Yang et al., 2015 [21]	n = 9/group male C57BL/6 mice	30 mg/kg MPTP i.p. 7 short	7 days	MWM	↓ latency
Yabuki et al., 2014 [22]	n = 10–11/group male C57BL/6N mice	25 mg/kg MPTP i.p. 5 short	28 days	Step-through passive avoidance task and NOR	↓ discrimination NOR
Castro et al., 2013 [23]	n = 6–7/group male Wistar rats	1 mg/nostril MPTP intranasally bilateral and	7 days = Social recognition 14 days = forced swimming, SPT 21 days = water maze	Social recognition, FST, SPT, and MWM	No ≠ social recognition, SPT and MWM. ↓ social recognition. ↑ FST

?: not specified in the study; short: indicates the number of doses on consecutive days; ↓: decrease; ↑: increase; ≠: difference; MPTP: 1-methyl-4-phenyl-1,2,3,6-tetrahydropyridine; MWM: Morris water maze test; FST: forced swimming test; i.p.: intraperitoneally; SPT: sucrose preference test; TST: tail suspension test; OFT: open field test; NOR: novel object recognition task.

**Table 3:** Characterization of experimental studies included in the review: 6-OHDA.

Author/ Year	Sample	Intervention Elements	Recovery Time	Measurement	Significant Results
Abrishamdar et al., 2023 [24]	n = 8/group male Wistar rats	20 µg/4 µL 6-OHDA into right MFB. 25 mg/kg of desipramine	2 weeks = rotation test 3 weeks = others	Apomorphine-induced rotation test, OFT, EPM, passive avoidance test and tail-flick test	↑ immobility OFT. ↓ EPM and passive avoidance. ↓ tail-flick test.
Antipova et al., 2021 [25]	n = 10–11/group male Wistar rats	24 μg 6-OHDA into right MFB	4 weeks	Apomorphine-Induced Rotation Test, amphetamine-Induced Rotation Test, OFT, EPM, FST and TST	= OFT, EPM and TST. ↑ frequency of immobility FST.
Bigham et al., 2021 [26]	n = 10/group male Wistar rats	2 µL/min 6-OHDA in the left MFB	2 weeks	Apomorphine-induced rotational test, OFT and passive avoidance test	↑ central zone OFT. ↓ latency passive avoidance.
Jalali et al., 2021 [27]	n = 10/group male Wistar rats	16 μg/2 μL 6-OHDA into right MFB. 15 mg/kg of desipramine	2 weeks = rotation test 4 months = others	Apomorphine-Induced Rotation Test, tail flick test, EPM and OFT	↓ tail flick latencies, EPM and OFT
Hamzehloei et al., 2019 [28]	n = 8/group male Wistar rats	16 μg/4 μL 6-OHDA into left MFB	6 weeks	Apomorphine-induced rotation test and passive avoidance memory	↓ latency passive avoidance
Haddadi et al., 2018 [29]	n = 7/group male Wistar rats	16 μg/4 μL 6-OHDA into the left MFB	2–6 weeks = rotation test 6 weeks = others	Apomorphine-induced rotations, passive avoidance memory and Tail-flick test	↓ latency passive avoidance and tail-flick
Souza et al., 2018 [30]	n = 10–12/group male Wistar rats	1 μL 6-OHDA into the SNpc	1–2–3 weeks	SPT	↓ sucrose
Turnes et al., 2018 [31]	n = 10–13/ group male Wistar rats	6 μg/μL 6-OHDA into SN	20–21 = NOR	NOR	↓ discrimination index
Hadadianpour et al., 2017 [32]	n = 7/group male Wistar rats	200 μg/5 μL 6-OHDA intracerebroventricul. 25 mg/kg desipramine	2 weeks	Passive avoidance learning	↓ latency
Singh et al., 2017 [33]	n = 6/group male Sprague– Dawley rats	2 μL 6-OHDA into right MFB	3 weeks	Light and Dark Test, social interaction test and FST	↓ time spent in the light. ↓ social interaction. ↑ immobility FST.
Ghiglieri et al., 2016 [34]	n = 6–8/group male Wistar rats	3 μg/μL 6-OHDA into the left MFB	2 weeks = rotation test 7 weeks = others	Apomorphine-induced rotation test, SPT and Active avoidance	↓ shock avoidance behavior.
Silva et al., 2016 [35]	n = 12/group male Wistar rats	12 μg 6-OHDA bilaterally into dorsal striatum. Desipramine	1–2–3 weeks	EPM and SPT	↓ fluid consumption SPT
Berg et al., 2015 [36]	n = 11–16/group male Wistar rats	6.5 μg 6-OHDA in the left MFB	1–3 weeks = Rotation test 3 weeks = other	D-Amphetamine Induced Rotation and eight-arm Radial maze	↓ time spent EPM and sucrose in SPT
Schwerk et al., 2015 [37]	n = 7/group male Wistar rats	2 μL 6-OHDA into the MFB	1–4 weeks = Rotation test 3 weeks = others	D-amphetamine-induced rotations and eight-arm radial maze	↓ short-term memory
Carmo et al., 2014 [38]	n = 8/group male Wistar rats	18 μg/3 μL 6-OHDA unilateral intrastriatal	15 to 18 days	Apomorphine-induced rotation test, passive avoidance test and MWM	↓ latency passive avoidance. ↓ performance MWM
Chen et al., 2014 [39]	n = 8–10/group male Wistar rats	12 µg/3 µL/side 6-OHDA bilaterally into the striatum	14 to 25 days	Cylinder test, Apomorphine-induced rotation test, EPM, SPT, social interaction and NOR	↓ EPM and sucrose SPT. = NOR. ↓ social interaction
Tuon et al., 2014 [40]	n = 12/group male C57BL mice	8 μg 6-OHDA unilateral in the terminal region of the striatum	1 week	Apomorphine-induced rotations and FST	↑ immobility FST
Kopalli et al., 2013 [41]	n = 6/group male C57BL/6N mice	10 μg 6-OHDA unilaterally into the SN. 25 mg/Kg desipramine	2 weeks	Apomorphine-induced rotational behavior test, passive avoidance test and Y-maze task	↓ latency passive avoidance. ↓ performance Y-maze

6-OHDA: 6-hydroxydopamine; MFB: medial forebrain bundle; EPM: elevated plus maze test; ↓: decrease; ↑: increase; =: no difference; SNpc: substantia nigra pars compacta; SN: substantia nigra; OFT: open field test; FST: forced swimming test; TST: tail suspension test; SPT: sucrose preference test; NOR: novel object recognition task.

**Table 4:** Characterization of experimental studies included in the review: rotenone.

Author/ Year	Sample	Intervention Elements	Recovery Time	Measurement	Significant Results
Madiha et al., 2021 [42]	n = 8/group male Wistar rats.	1.5 mg/kg rotenone subcutaneously 8 short	2 weeks	Social interaction test, SPT, NOR and MWM	↓ interaction social. ↓ sucrose SPT. ↓ NOR. ↑ escape latency MWM
Zhao et al., 2021 [43]	n = 8–12/group male C57BL/6J mice.	2.5 mg/kg rotenone i.p. 14 short	1 day = include 4 weeks = others	TST and SPT	↑ immobility TST. ↓ sucrose SPT
Haider et al., 2020 [44]	n = 6/group Wistar rats.	1.5 mg/kg rotenone s.c. 8 short	3 weeks	MWM and NOR	↑ escape latency MWM. ↓ preference index NOR
Medeiros-Linard et al., 2018 [45]	n = 8/group male Wistar rats.	3 mg/kg/day rotenone s.c. 5 shorts	1 day	OFT and T-maze test	↑ immobility OFT. ↓ memory retention T-maze

Short: indicates the number of doses on consecutive days; s.c.: subcutaneously; SPT: sucrose preference test; NOR: novel object recognition task; MWM: Morris water maze; ↓: decrease; ↑: increase; TST: tail suspension test; OFT: open field test.

## Data Availability

All data generated or analyzed during this study are included in this published article. No primary data were collected, as this study is based on previously published literature.

## References

[1] Simon D.K., Tanner C.M., Brundin P. (2020). Parkinson Disease Epidemiology, Pathology, Genetics, and Pathophysiology. Clin. Geriatr. Med..

[2] Raza C., Anjum R., Shakeel N.U.A. (2019). Parkinson’s disease: Mechanisms, translational models and management strategies. Life Sci..

[3] Galet B., Ingallinesi M., Pegon J., Thi A.D., Ravassard P., Biguet N.F., Meloni R. (2021). G-protein coupled receptor 88 knockdown in the associative striatum reduces psychiatric symptoms in a translational male rat model of Parkinson disease. J. Psychiatry Neurosci..

[4] Murueta-Goyena A., Andikoetxea A., Gómez-Esteban J.C., Gabilondo I. (2019). Contribution of the GABAergic System to Non-Motor Manifestations in Premotor and Early Stages of Parkinson’s Disease. Front. Pharmacol..

[5] Tansey M.G., Wallings R.L., Houser M.C., Herrick M.K., Keating C.E., Joers V. (2022). Inflammation and immune dysfunction in Parkinson disease. Nat. Rev. Immunol..

[6] Ray Chaudhuri K., Poewe W., Brooks D. (2018). Motor and Nonmotor Complications of Levodopa: Phenomenology, Risk Factors, and Imaging Features. Mov. Disord..

[7] Prasad E.M., Hung S.Y. (2020). Behavioral Tests in Neurotoxin-Induced Animal Models of Parkinson’s Disease. Antioxidants.

[8] Zhang T.D., Kolbe S.C., Beauchamp L.C., Woodbridge E.K., Finkelstein D.I., Burrows E.L. (2022). How Well Do Rodent Models of Parkinson’s Disease Recapitulate Early Non-Motor Phenotypes? A Systematic Review. Biomedicines.

[9] Quiroga-Varela A., Aguilar E., Iglesias E., Obeso J.A., Marin C. (2017). Short- and long-term effects induced by repeated 6-OHDA intraventricular administration: A new progressive and bilateral rodent model of Parkinson’s disease. Neuroscience.

[10] Vieira J.C.F., Bassani T.B., Santiago R.M., Guaita G.d.O., Zanoveli J.M., da Cunha C., Vital M.A. (2019). Anxiety-like behavior induced by 6-OHDA animal model of Parkinson’s disease may be related to a dysregulation of neurotransmitter systems in brain areas related to anxiety. Behav. Brain Res..

[11] Branchi I., D’ANdrea I., Armida M., Cassano T., Pèzzola A., Potenza R.L., Morgese M.G., Popoli P., Alleva E. (2008). Nonmotor symptoms in Parkinson’s disease: Investigating early-phase onset of behavioral dysfunction in the 6-hydroxydopamine-lesioned rat model. J. Neurosci. Res..

[12] Carnicella S., Drui G., Boulet S., Carcenac C., Favier M., Duran T., Savasta M. (2014). Implication of dopamine D3 receptor activation in the reversion of Parkinson’s disease-related motivational deficits. Transl. Psychiatry.

[13] Hooijmans C.R., Rovers M.M., de Vries R.B.M., Leenaars M., Ritskes-Hoitinga M., Langendam M.W. (2014). SYRCLE’s risk of bias tool for animal studies. BMC Med. Res. Methodol..

[14] Yang X., Zhao Y., Liang L., Qu Y., Yu C., Zhang J., Lian W., Zhao Y. (2025). Protective effect of ginsenoside CK against MPTP-induced Parkinson’ s disease mouse model by suppressing oxidative stress and inflammation, and modulating the gut microbiota. Microb. Pathog..

[15] Yang C., Song Y., Luo M., Wang Q., Zhang Y., Cen J., Du G., Shi J. (2025). Exosomes-encapsulated biomimetic polydopamine carbon dots with dual-targeting effect alleviate motor and non-motor symptoms of Parkinson’s disease via anti-neuroinflammation. Int. J. Biol. Macromol..

[16] Liu Y., Zhou X., Xue K., Sun R., Tang Y., Tang C. (2024). Reviving: Restoring depression-like behaviour through glial cell-derived neurotrophic factor treatment in the medial prefrontal cortex. J. Psychiatry Neurosci..

[17] Datta I., Mekha S.R., Kaushal A., Ganapathy K., Razdan R. (2020). Influence of intranasal exposure of MPTP in multiple doses on liver functions and transition from non-motor to motor symptoms in a rat PD model. Naunyn Schmiedebergs Arch. Pharmacol..

[18] Haga H., Yamada R., Izumi H., Shinoda Y., Kawahata I., Miyachi H., Fukunaga K. (2020). Novel fatty acid-binding protein 3 ligand inhibits dopaminergic neuronal death and improves motor and cognitive impairments in Parkinson’s disease model mice. Pharmacol. Biochem. Behav..

[19] Tang J., Lu L., Wang Q., Liu H., Xue W., Zhou T., Xu L., Wang K., Wu D., Wei F. (2020). Crocin Reverses Depression-Like Behavior in Parkinson Disease Mice via VTA-mPFC Pathway. Mol. Neurobiol..

[20] Hsieh M.-H., Meng W.-Y., Liao W.-C., Weng J.-C., Li H.-H., Su H.-L., Lin C.-L., Hung C.-S., Ho Y.-J. (2017). Ceftriaxone reverses deficits of behavior and neurogenesis in an MPTP-induced rat model of Parkinson’s disease dementia. Brain Res. Bull..

[21] Yang W., Chen Y.H., Liu H., Qu H.D. (2015). Neuroprotective effects of piperine on the 1-methyl-4-phenyl-1,2,3,6-tetrahydropyridine-induced Parkinson’s disease mouse model. Int. J. Mol. Med..

[22] Yabuki Y., Ohizumi Y., Yokosuka A., Mimaki Y., Fukunaga K. (2014). Nobiletin treatment improves motor and cognitive deficits seen in MPTP-induced Parkinson model mice. Neuroscience.

[23] Castro A.A., Wiemes B.P., Matheus F.C., Lapa F.R., Viola G.G., Santos A.R., Tasca C.I., Prediger R.D. (2013). Atorvastatin improves cognitive, emotional and motor impairments induced by intranasal 1-methyl-4-phenyl-1,2,3,6-tetrahydropyridine (MPTP) administration in rats, an experimental model of Parkinson’s disease. Brain Res..

[24] Abrishamdar M., Farbood Y., Sarkaki A., Rashno M., Badavi M. (2023). Evaluation of betulinic acid effects on pain, memory, anxiety, catalepsy, and oxidative stress in animal model of Parkinson’s disease. Metab. Brain Dis..

[25] Antipova V., Holzmann C., Hawlitschka A., Witt M., Wree A. (2021). Antidepressant-Like Properties of Intrastriatal Botulinum Neurotoxin-A Injection in a Unilateral 6-OHDA Rat Model of Parkinson’s Disease. Toxins.

[26] Bigham M., Mohammadipour A., Hosseini M., Malvandi A.M., Ebrahimzadeh-Bideskan A. (2021). Neuroprotective effects of garlic extract on dopaminergic neurons of substantia nigra in a rat model of Parkinson’s disease: Motor and non-motor outcomes. Metab. Brain Dis..

[27] Jalali M.S., Saki G., Farbood Y., Azandeh S.S., Mansouri E., Dehcheshmeh M.G., Sarkaki A. (2021). Therapeutic effects of Wharton’s jelly-derived Mesenchymal Stromal Cells on behaviors, EEG changes and NGF-1 in rat model of the Parkinson’s disease. J. Chem. Neuroanat..

[28] Hamzehloei L., Rezvani M.E., Rajaei Z. (2019). Effects of carvacrol and physical exercise on motor and memory impairments associated with Parkinson’s disease. Arq. Neuropsiquiatr..

[29] Haddadi H., Rajaei Z., Alaei H., Shahidani S. (2018). Chronic treatment with carvacrol improves passive avoidance memory in a rat model of Parkinson’s disease. Arq. Neuropsiquiatr..

[30] Souza L.C., Martynhak B.J., Bassani T.B., Turnes J.d.M., Machado M.M., Moura E., Andreatini R., Vital M.A. (2018). Agomelatine’s effect on circadian locomotor rhythm alteration and depressive-like behavior in 6-OHDA lesioned rats. Physiol. Behav..

[31] Turnes J.M., Bassani T.B., Souza L.C., Vital M.A.B.F. (2018). Ineffectiveness of saxagliptin as a neuroprotective drug in 6-OHDA-lesioned rats. J. Pharm. Pharmacol..

[32] Hadadianpour Z., Fatehi F., Ayoobi F., Kaeidi A., Shamsizadeh A., Fatemi I. (2017). The effect of orexin-A on motor and cognitive functions in a rat model of Parkinson’s disease. Neurol. Res..

[33] Singh S., Mishra A., Srivastava N., Shukla S. (2017). MK-801 (Dizocilpine) Regulates Multiple Steps of Adult Hippocampal Neurogenesis and Alters Psychological Symptoms via Wnt/β-Catenin Signaling in Parkinsonian Rats. ACS Chem. Neurosci..

[34] Ghiglieri V., Mineo D., Vannelli A., Cacace F., Mancini M., Pendolino V., Napolitano F., di Maio A., Mellone M., Stanic J. (2016). Modulation of serotonergic transmission by eltoprazine in L-DOPA-induced dyskinesia: Behavioral, molecular, and synaptic mechanisms. Neurobiol. Dis..

[35] Silva T.P., Poli A., Hara D.B., Takahashi R.N. (2016). Time course study of microglial and behavioral alterations induced by 6-hydroxydopamine in rats. Neurosci. Lett..

[36] Berg J., Roch M., Altschüler J., Winter C., Schwerk A., Kurtz A., Steiner B. (2015). Human adipose-derived mesenchymal stem cells improve motor functions and are neuroprotective in the 6-hydroxydopamine-rat model for Parkinson’s disease when cultured in monolayer cultures but suppress hippocampal neurogenesis and hippocampal memory function when cultured in spheroids. Stem Cell Rev. Rep..

[37] Schwerk A., Altschüler J., Roch M., Gossen M., Winter C., Berg J., Kurtz A., Akyüz L., Steiner B. (2015). Adipose-derived human mesenchymal stem cells induce long-term neurogenic and anti-inflammatory effects and improve cognitive but not motor performance in a rat model of Parkinson’s disease. Regen. Med..

[38] Carmo M.R., Menezes A.P.F., Nunes A.C.L., Pliássova A., Rolo A.P., Palmeira C.M., Cunha R.A., Canas P.M., Andrade G.M. (2014). The P2X7 receptor antagonist Brilliant Blue G attenuates contralateral rotations in a rat model of Parkinsonism through a combined control of synaptotoxicity, neurotoxicity and gliosis. Neuropharmacology.

[39] Chen L., Deltheil T., Turle-Lorenzo N., Liberge M., Rosier C., Watabe I., Sreng L., Amalric M., Mourre C. (2014). SK channel blockade reverses cognitive and motor deficits induced by nigrostriatal dopamine lesions in rats. Int. J. Neuropsychopharmacol..

[40] Tuon T., Valvassori S., Pont G.D., Paganini C., Pozzi B., Luciano T., Souza P., Quevedo J., Souza C., Pinho R. (2014). Physical training prevents depressive symptoms and a decrease in brain-derived neurotrophic factor in Parkinson’s disease. Brain Res. Bull..

[41] Kopalli S.R., Noh S.J., Koppula S., Suh Y.H. (2013). Methylparaben protects 6-hydroxydopamine-induced neurotoxicity in SH-SY5Y cells and improved behavioral impairments in mouse model of Parkinson’s disease. Neurotoxicology.

[42] Madiha S., Batool Z., Tabassum S., Liaquat L., Sadir S., Shahzad S., Naqvi F., Saleem S., Yousuf S., Nawaz A. (2021). Quercetin exhibits potent antioxidant activity, restores motor and non-motor deficits induced by rotenone toxicity. PLoS ONE.

[43] Zhao X., Kong D., Zhou Q., Wei G., Song J., Liang Y., Du G. (2021). Baicalein alleviates depression-like behavior in rotenone- induced Parkinson’s disease model in mice through activating the BDNF/TrkB/CREB pathway. Biomed. Pharmacother..

[44] Haider S., Madiha S., Batool Z. (2020). Amelioration of motor and non-motor deficits and increased striatal APoE levels highlight the beneficial role of pistachio supplementation in rotenone-induced rat model of PD. Metab. Brain Dis..

[45] Medeiros-Linard C.F.B., Andrade-Da-Costa B.L.d.S., Augusto R.L., Sereniki A., Trevisan M.T.S., Perreira R.d.C.R., de Souza F.T.C., Braz G.R.F., Lagranha C.J., de Souza I.A. (2018). Anacardic Acids from Cashew Nuts Prevent Behavioral Changes and Oxidative Stress Induced by Rotenone in a Rat Model of Parkinson’s Disease. Neurotox. Res..

[46] Vingill S., Connor-Robson N., Wade-Martins R. (2018). Are rodent models of Parkinson’s disease behaving as they should?. Behav. Brain Res..

[47] Yin R., Zhang K., Li Y., Tang Z., Zheng R., Ma Y., Chen Z., Lei N., Xiong L., Guo P. (2023). Lipopolysaccharide-induced depression-like model in mice: Meta-analysis and systematic evaluation. Front. Immunol..

[48] Kaluve A.M., Le J.T., Graham B.M. (2022). Female rodents are not more variable than male rodents: A meta-analysis of preclinical studies of fear and anxiety. Neurosci. Biobehav. Rev..

